# A Retrospective Evaluation and Review of Outcomes for Single- and Multilevel ACDF With a Zero-Profile Stand-Alone Cage Device With Integrated Instrumentation

**DOI:** 10.7759/cureus.14283

**Published:** 2021-04-03

**Authors:** Kareem Khalifeh, Jordan E Faulkner, Junko Hara, Burak Ozgur

**Affiliations:** 1 Neurosurgery, ONE Brain and Spine Center, Newport Beach, USA; 2 Neurosurgery, Hoag Memorial Hospital, Newport Beach, USA; 3 Neurosurgery, ONE Brain and Spine Center, Irvine, USA; 4 Neurosurgery, Pickup Family Neurosciences Institute, Newport Beach, USA

**Keywords:** stand-alone, zero-profile, anterior cervical discectomy and fusion, acdf

## Abstract

This study aimed to assess clinical and radiological outcomes associated with zero-profile stand-alone cages with instrumentation used for single- and multilevel anterior cervical discectomy and fusion (ACDF) operations. Many plate-cage ACDF systems have proven to be successful in producing high fusion rates and positive clinical outcomes. However, the anterior plating in traditional systems has been associated with complications such as dysphagia and mechanical accidents. A total of 190 patients underwent single- or multilevel ACDF surgeries with zero-profile polyetheretherketone cages containing integrated titanium instrumentation and screw fixation (one-level, n=31; two-level, n=65; three-level, n=71; four-level, n=23). Demographic information such as age and smoking status as well as postoperative outcomes were collected and analysed. Out of the 190 patients who underwent ACDF surgeries with a zero-profile stand-alone cage, none experienced any conditions or infections, and zero were readmitted postoperatively. Although traditional plate-cage systems yield high fusion rates in ACDF surgeries, zero-profile systems with integrated fixation have showcased impressive clinical and radiographic results in both single- and multilevel operations.

## Introduction

The anterior surgical approach to the cervical spine introduced by Smith and Robinson has become a widely accepted strategy and one of the most commonly performed spinal procedures in the neuro-orthopedic world [1]. It is also one of the most rewarding and efficient spinal operations, offering relatively easy access to the vertebral column and satisfactory outcomes in most cases [2]. Furthermore, anterior cervical discectomy and fusion (ACDF) has become accepted as the leading method for treating patients with degenerative disc disease by promoting vertebral fusion, decompressing the spinal cord and nerve roots, restoring lordosis and maintaining intervertebral disc height [3].

Many traditional plate-cage ACDF systems have produced successful fusion rates and positive clinical outcomes in single-level operations, so similar techniques have been adopted in multilevel procedures as well where the likelihood of fusion is lower [4]. However, although anterior plating in an ACDF structure may promote fusion and maintain proper vertebral alignment, it is also associated with potential plate-related complications such as dysphagia, pseudarthrosis, plate movement or migration, and longer operative times, especially in multilevel procedures [5-8]. Stand-alone cages without a plate have also been used due to their less invasive nature; however, greater risks of cage subsidence and lower fusion rates have been associated with this model [9].

Recently, our experience has shown that the use of stand-alone polyetheretherketone (PEEK) cages with integrated instrumentation and screw fixation has been associated with satisfactory fusion rates in both single- and multilevel operations. We have also showcased that the zero-profile system minimizes complication and infection rates while providing efficient operations.

The purpose of this study was to assess clinical and radiological outcomes associated with zero-profile stand-alone cages with instrumentation used for single- and multilevel ACDF and evaluate the efficiency and practicality of the surgical technique.

## Materials and methods

Study design

This was a retrospective review involving clinical and radiological outcomes of 190 adult patients who underwent single- or multilevel ACDF surgeries with zero-profile PEEK cages with instrumentation and plate fixation between January 2014 and December 2019. All ACDF procedures were performed using a similar technique by a single surgeon. Patient demographics collected included age, gender and smoking status. Patients were assessed preoperatively with the visual analog scale (VAS) and postoperatively at two-week, six-week, three-month, six-month, and one-year follow-ups. Patients received X-ray imaging starting at the three-month interval in order to evaluate fusion progress and instrumentation placement.

Inclusion criteria included the following: (I) adult patients over the age of 18 years old, (II) degenerative disc disease causing symptomatic cervical radiculopathy confirmed by magnetic resonance imaging (MRI) and clinical assessments and (III) patients undergoing single- or multilevel ACDF with zero-profile stand-alone cage system with integrated instrumentation and screw fixation. Exclusion criteria were as follows: (I) active malignancy or infection, (II) allergy to cage materials, (III) trauma, (IV) severe osteoporosis and (V) pregnancy. This study was approved by our institutional review board at Hoag Hospital Newport Beach, California.

Surgical procedure

**Figure 1:**
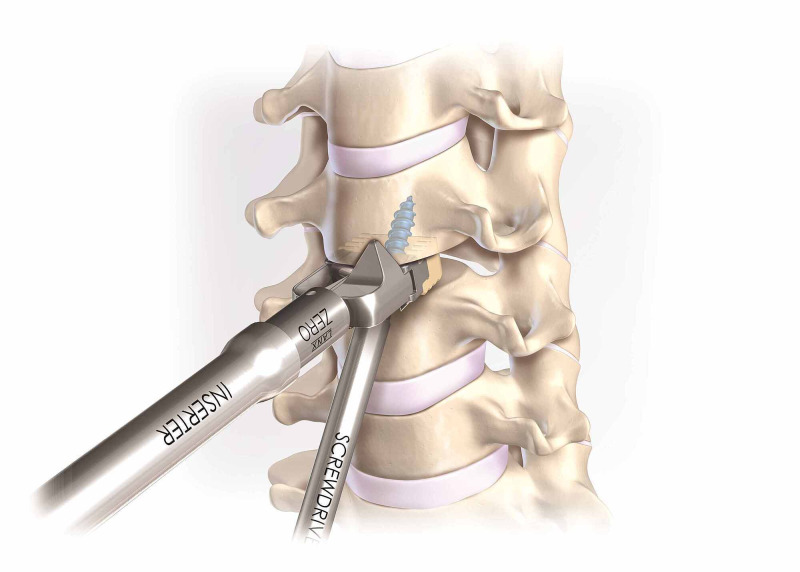
Drawing of the manual screwdriver inserting screw through the polyetheretherketone cage and into the vertebral body

All surgical procedures were executed using a Smith-Robinson approach under general anesthesia. After initial incision and tissue dissection, a spinal needle was inserted into the disc space of interest, and its location was confirmed using C-arm digital radiography. Retraction pins were then implanted into the vertebral bodies to allow for the insertion of implants. Disc material, cartilage and unnecessary bone and tissue were removed using a combination of osteotomes, curettes, rasps and rongeurs. Trials were inserted into the intervertebral disc space to determine the size of the desired zero-profile cage implant. The PEEK cage system was packed with autologous bone graft or allograft to promote bone development and fusion and was positioned into the disc space. An awl or drill was inserted into the screw pockets on the anterior of the cage and into the bone, and a freehand screwdriver was used to place the screws into the prepared screw holes (Figure 1). Locking caps were inserted to prevent the screws from falling out. The retraction pins were removed, and the discectomy and cage-insertion process repeated for the multilevel operations. Finally, the fascia and skin incision were closed.

Implant characteristics

**Figure 2:**
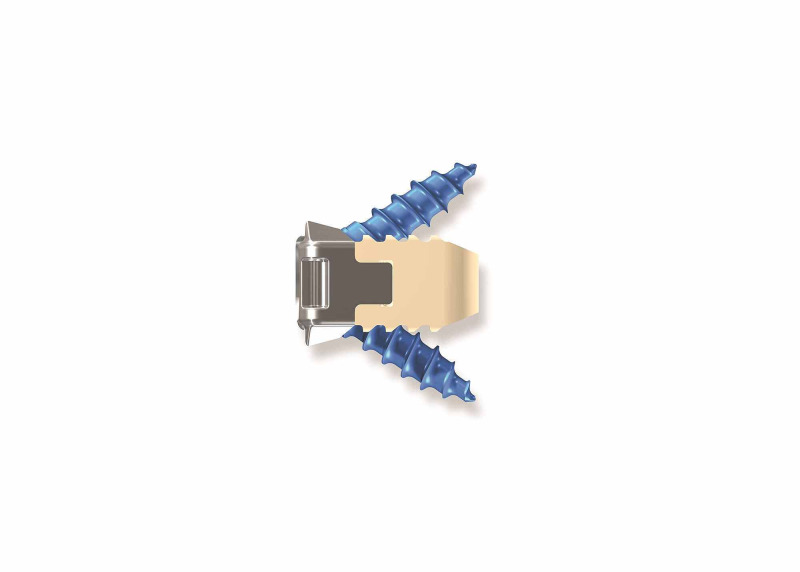
Drawing of a zero-profile cage implant

The zero-profile stand-alone screw/cage system (Figure 2) sits entirely inside of the interbody space to minimize contact with the esophagus. The small anterior plate sits flush in between the vertebral bodies, and the two titanium screws that position inside of the vertebral bodies provide fixation while enabling an efficient surgical operation. Each screw is covered by a locking cap that prevents it from backing out. The PEEK cage itself is rounded with various heights and footprints. It also has a central cavity to accommodate the bone graft. The upper and lower surfaces have sequences of transverse grooves to improve stability once inserted.

## Results

A total of 190 adult patients (99 males, 91 females) participated in this study (Table 1). The average age of patients was 66 years old, and the majority (n=136) of patients were non-smokers. A total of 466 implants/devices were used in the study. A total of 31 patients underwent single-level operations, 65 underwent two-level, 71 underwent three-level and 23 underwent four-level operations (Table 2). The average length of stay after the operation was one day. In the first several weeks following an ACDF, it is very common to have temporary mild complaints of neck/shoulder pain, soreness, and muscle spams as well as mild temporary dysphagia. These temporary symptoms resolved within the first month after surgery that is usual and expected and not considered a complication. Out of the 190 patients, none experienced any complications, including chronic dysphagia or functional pseudarthrosis, or infections, and no readmissions were directed. We also received no complaints about chronic radicular pain, neck pain or disability.

**Table 1:** Patient demographics

Patient Demographics	Number of Patients
Gender	
Male	99
Female	91
Age range	
20-40 years	6
40-60 years	61
60-80 years	111
90+ years	12
Average	66
Smoking status	
Current smoker	7
Non-smoker	136
Former smoker	33
Unknown	14

**Table 2:** Single- and Multilevel cases

Number of Levels	Number of Patients
1 level	31
2 levels	65
3 levels	71
4 levels	23

All patients were urged for postoperative follow-ups at two weeks, six weeks, three months, six months and one year in which they received X-ray imaging starting at the six-week follow-up interval. As the duration of postoperative follow-up lengthened, less patients chose to return due to reported satisfaction and although some patients were lost before they could complete their follow-up, no complaints were reported.

Corpectomy cases and patients requiring anterior/posterior combined surgical techniques were excluded from this analysis and data as to be able to more fairly compare published traditional ACDF data.

## Discussion

Our data has demonstrated that zero-profile stand-alone cage systems with integrated instrumentation showcase successful results when used for ACDF. The screw/cage system demonstrated ideal functionality in both single-level and multilevel operations. All procedures resulted in spinal cord decompression, effective vertebral fusion and lordosis restoration, relieving patients of radicular pain and/or disability. Remarkably, no patients experienced any problems during the procedures nor any postoperative complications including functional pseudarthrosis, subsidence or chronic dysphagia. Comparatively, studies have demonstrated that plate-related complications in traditional plate-cage systems are high. For instance, Fisahn et al. reported plate-related dysphagia at 12.1% whereas Segebarth et al. reported it to be as high as 42.1% [6,10]. High chronic dysphagia rates and other plate-related complications have provoked the development of new surgical techniques to ultimately improve short- and long-term patient outcomes. As a result, stand-alone cages without instrumentation were developed, but they presented issues regarding subsidence and fusion rates. Ng et al. highlighted the model’s subsidence rate to be 22.5% [11]. We propose that zero-profile cage systems with instrumentation and screw fixation should be considered viable options for ACDF due to their effective design and promising results.

## Conclusions

The minimally invasive nature of the screw/cage system avoids unnecessary contact with the esophagus to help minimize dysphagia. Additionally, the angled screw insertion helps anchor the cage within the intervertebral space, while the transverse grooves of the cage help prevent the vertebrae from collapsing or slipping.

This study demonstrates that compared to traditional plate-cage systems, zero-profile cages with instrumentation provide efficient operations and facilitate fusion across both single- and multilevel ACDFs. This demonstrates an acceptable and preferable option for ACDFs in many cases.
